# Study on Electrochemical Corrosion Behavior of Plasma Sprayed Al_2_O_3_-3%TiO_2_ Coatings Doped with CeO_2_ for Long-Term Immersion

**DOI:** 10.3390/ma18194532

**Published:** 2025-09-29

**Authors:** Jiahang Yan, Yu Zhang, Pengyu Dai, Lin Zhao, Xin Wang, Xiaohong Yi

**Affiliations:** 1 School of Mechanical Engineering, Shenyang University, Shenyang 110044, China; 13470377670@163.com (J.Y.); nihao9985@163.com (Y.Z.);; 2 Institute of Metal Research, Chinese Academy of Sciences, Shenyang 110016, China; 3 Engineering Center for Superlubricity, Jihua Laboratory, Foshan 528000, China

**Keywords:** Al_2_O_3_-TiO_2_ coating, the content of CeO_2_, long-term immersion, valence of Ce

## Abstract

The long-term corrosion behavior of Al_2_O_3_-3%TiO_2_ (AT3) coatings doped with1%, 5% and 8% CeO_2_ prepared by plasma spraying was studied in 5% NaCl solution. The results showed that the protective performance of CeO_2_-doped coatings was significantly higher than that of undoped coatings, primarily due to the reduction in coating porosity caused by the addition of rare-earth elements. Among the doped coatings, the 5% CeO_2_-doped coating exhibited the best protective performance. The addition of rare-earth oxides CeO_2_ reduced the content of γ-Al_2_O_3_ in the coating, but when the concentration of CeO_2_ increased to 8%, the Ce element was rich in the gap of the coating. Excessive CeO_2_ enriched in the gaps and coexisted more with Ti, and prevented the formation of the AlTi phase, which affected the performance of the coating. Electrochemical and XPS results revealed that an appropriate amount of Ce atoms or CeO_2_ particles could fill the pores of the coating. During long-term immersion, Ce (IV) was converted to Ce (III), which demonstrated that Ce atoms have high chemical activity in coatings. The thermodynamic calculation results show that more CeO_2_ particles improved the adsorption of corrosive ions. It indicated that the content of doped rare-earth oxides exceeding 5% would be utilized as an active material in the corrosive process.

## 1. Introduction

Al_2_O_3_-TiO_2_ coating is a ceramic coating with high resistance to heat, corrosion, and wear, as well as low cost, good chemical stability, and excellent wear resistance, which extends the service life of components [1]. It is widely employed in the nuclear industry, petrochemical industry, electronics industry, and other sectors [2,3,4]. There are several techniques to prepare Al_2_O_3_-TiO_2_ coating, such as Sol–Gel [5], CVD [6], laser engraving [7], and plasma spraying. Among these, plasma spraying is popular for fabricating various functional coatings [8,9]. Due to its numerous benefits, including high spraying temperature, high deposition rate, minimal heat effect on the substrate, and a wide range of material options, plasma spray has become a significant surface engineering technology frequently used for the surface repairing and strengthening of mechanical parts [10,11].

Most of the benefits of the Al_2_O_3_-TiO_2_ coating still remain after it has been created via plasma spraying; nevertheless, the coating’s porosity and flaws affect how well it resists corrosion [3,12]. Therefore, many techniques were applied, including heat treatment, sealing, doping rare-earth elements, doping nanoparticles, and nanoscale powders, to reduce the porosity and flaws of the coating [3,13,14,15]. Among these techniques, doping with rare-earth oxides into the coating has proven to be an effective method for reducing its porosity and flaws. Rare-earth oxides typically occupy the inter-splat boundaries and pores of the coating, thereby enhancing its corrosion resistance [16,17]. In our earlier research [18], it was found that the rare-earth oxide CeO_2_ decreased the coating’s porosity and enhanced protectiveness of the Al_2_O_3_-TiO_2_ coating. The coating doped with 5 wt% CeO_2_ showed a more compact structure, which prevented chloride ion penetration, and improved its protectiveness. However, the question remains whether further increasing the content of rare-earth oxides in the coating can enhance its qualities, particularly in terms of long-term corrosion resistance. There are now several studies on coating with Al_2_O_3_-TiO_2_ or doping with rare-earth oxide, and many studies on its corrosion behavior [16,19,20], but research on the long-term corrosion behavior of such coatings remains limited. In long-term immersion for the coating prepared by plasma spraying, defects such as pores and cracks become an effective channel for corrosive ions to penetrate the coating, causing a fatal impact on the oxide coating. This process obviously reduces the isolation and shielding effect of the coating, directly causing corrosion to the protected matrix. As an effective method to improve the porosity of the coating, the role of rare-earth elements in the long-term immersion process remains to be further studied.

In this work, an Al_2_O_3_-3%TiO_2_ coating was created on carbon steel (Q235B) by plasma spraying, and different amounts of the rare-earth oxide CeO_2_ (1%, 5%, 8%) were added. All of the coatings were immersed in 5% NaCl solution for 48 h and monitored by electrochemical methods to evaluate the effect of the rare-earth oxide CeO_2_ during long-term immersion. This work uniquely quantifies Ce^4+^→Ce^3+^ conversion through XPS depth profiling (36 h immersion) and establishes its correlation with electrochemical degradation, and then determines the upper doping limit and long-term electrochemical stability. The valence state changes in different concentrations of CeO_2_ in the coating during the long-term immersion process, and its role in the corrosion process was investigated in an effort to find the optimal proportion of rare-earth oxide CeO_2_ in the coating, which provides the first upper doping limit (5 wt%) for long-term stability. The corrosion behavior of the coating during long-term immersion was discussed by thermodynamic calculation.

## 2. Experimental

The substrate was carbon steel Q235B with a dimension of 20 mm by 15 mm by 3 mm in size. The chemical composition of Q235B (wt. %) is C 0.20, S 0.036, P 0.017, Mn 0.58, Si 0.21, Cu 0.02, and residual Fe. In order to create a rough surface and enough contact points for the sprayed coatings, the substrate was degreased with acetone before spraying.

Shanghai Koting Machinery Technology Co., Ltd. (Shanghai, China) manufactured the spray powder. The quality of the product is 99.9% Al_2_O_3_-3% TiO_2_ (mass fraction) mixed powder (referred to as AT3) and 99.5% CeO_2_ powder. The CeO_2_ powder is a lamellar strip with an average diameter of around 20 μm, and the particle length after mixing is 5 μm with a diameter of less than 1 μm.

The appropriate quantities of AT3 and CeO_2_ powder were combined with alcohol, and the mixing ratio is presented in Table 1. The mass ratio of alcohol to powder was 3:1, and FS400W high-speed dispersion mechanical stirring (400 RPM) was used on the powder for 60 min after ultrasonic dispersion for 30 min. After mixing, the resulting mixture was put into a drying oven set at 50 °C for 72 h to create a powder that is a combination of AT3 and CeO_2_. In Table 1, the concentration of Ce is the proportion of powder composition during powder mixing, which is the theoretical data. During the spraying process, the distribution of cerium oxide was not uniform due to powder melting, sputtering, and solidification, and the concentration of cerium oxide in the gaps and defects was high.

The particle size was around 20–50 μm, and it was demonstrated that the surface of the AT3 powder to be sprayed was uniformly covered with a layer of rare-earth oxide CeO_2_. An SX-80 plasma spraying equipment (Guangzhou Sanxin Metal Technology Co. Ltd., Guangzhou, China) was used to spray the AT3-CeO_2_ composite and AT3 powder, which includes a main power supply, chiller, powder feeder, transfer box, control cabinet, and SG-100 spray gun. Table 2 shows the spraying process parameters and the principal gas used. Reciprocating automatic spray gun operation mode was adopted, where the operation speed is 15 mm s^−1^, the thickness of each layer is about 15 μm, and a total of 4 layers are sprayed. The coating was cooled naturally after preparation without other post-treatment.

The coating adhesion test was conducted on the Chinese national standard “GB 5270 Metallic coatings on metallic substrates—Electrodeposited and chemically deposited coatings—Review of adhesion testing methods.” After three thermal shock tests at 400 °C, the coating remained undamaged.

The ceramic coating had a thickness of 50–70 µm, which was measured at 10 random locations per sample using SEM cross-sectional analysis (ImageJ v1.53 software, Bethesda, USA). A scanning electron microscope (SEM, S-4800II, Hitachi, Tokyo, Japan) was used to examine the as-sprayed coating surface and cross-sectional morphology. The phase of the coating was determined using a Rigaku-D/max 2000 (Rigaku Corporation, Tokyo, Japan) diffractometer with a Cu K target and a power range of 50 kv−250 mA and a scanning speed of 2°/min.

The chemical composition of the coatings was analyzed with a Thermo VG ESCALAB 250 X-ray photoelectron spectroscopy (XPS) (Thermo Fisher, Waltham, MA, USA). A monochromatic Al Ka was used as the X-ray source (incident energy of 1486.6 eV, pass energy of 50 eV), and it is powered at 150 W. The sample was etched by ionized argon at a vacuum of 6 × 10^−8^ mbar and an etching rate of 0.2 nm/s. The etched area was 2 mm × 2 mm. The binding energy (BE) in the XPS spectra was calibrated according to the standard BE of the C 1s peak (284.6 eV).

Surface hydrophobicity and chemical bonding analysis (e.g., FTIR) were not performed due to the focus on electrochemical and microstructural corrosion mechanisms.

### Electrochemical Test

On the reverse side of the coated Q235B substrate, a copper wire was attached. After thermal spraying, the samples were encased in Teflon and paraffin, exposing just a 1 cm^2^ coated area. The samples were cleaned with distilled water, degreased with acetone, and dried with a jet of air before the corrosion test. The Gamry reference 600 electrochemical workstation (Gamry Instruments, Warminster, PA, USA) was used for the electrochemical tests, and measurements were performed using the standard three-electrode system: the coated sample as the working electrode, the Pt electrode as the auxiliary electrode (CE), and the saturated calomel electrode (SCE) as the reference electrode. Corrosion potential (Ecorr) and the corrosion current density (Icorr) were obtained through the linear analysis of the Tafel fitting approximation.

The potentiodynamic polarization test had a scanning range of −0.2 V~0.8 V relative to open-circuit potential. The scanning rate was one millivolt per second. The electrochemical impedance spectroscopy (EIS) test used a sinusoidal AC signal with an amplitude of 10 mV and a scanning frequency ranging from 10^5^ to 10^−2^ Hz. The Zview software (E Chem software, V3.1, MI, USA) was used to fit the impedance spectra acquired by the EIS test. All the tests were repeated five times to confirm the accuracy of the results. At room temperature (18 °C), 5% NaCl (mass fraction) was utilized as the test solution. To ensure the reproducibility of experiments and the accuracy of data, electrochemical tests were conducted five times each.

## 3. Results and Discussion

### 3.1. SEM

**Figure 1:**
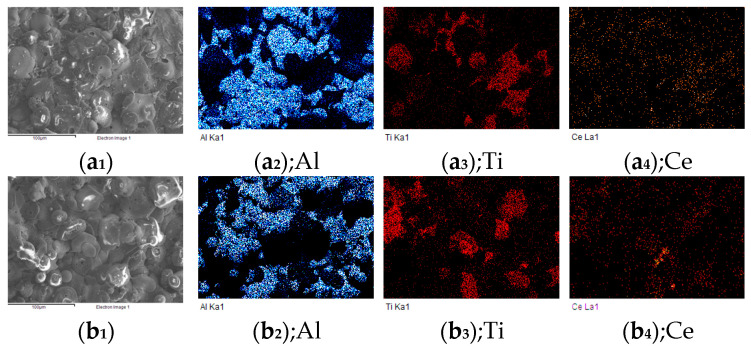

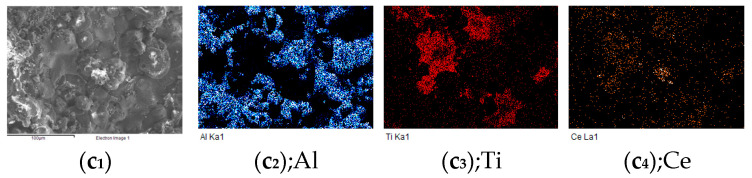
Element distribution maps of AT3 doped with 1%, 5%, 8% CeO_2_ coatings: a. Element distribution maps of AT3 + 1% CeO_2_ coating—(**a_1_**) surface morphology of AT + 1% CeO_2_ coating, (**a_2_**) Al element distribution maps, (**a_3_**) Ti element distribution maps, and (**a_4_**) Ce element distribution maps; b. Element distribution maps of AT3 + 5% CeO_2_ coating—(**b_1_**) surface morphology of AT3 + 5% CeO_2_ coating, (**b_2_**) Al element distribution maps, (**b_3_**) Ti element distribution maps, and (**b_4_**) Ce element distribution maps; c. Element distribution maps of AT3 + 8% CeO_2_ coating—(**c_1_**) surface morphology of AT3 + 8% CeO_2_ coating, (**c_2_**) Al element distribution maps, (**c_3_**) Ti element distribution maps, and (**c_4_**) Ce element distribution maps.

**Figure 2:**
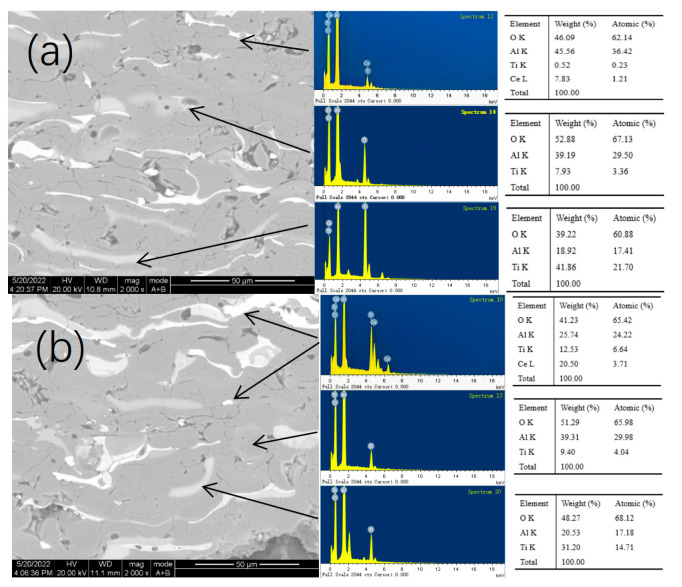
Figures and element distribution of cross-section morphology for AT3 doped with 5%, 8% CeO_2_ coatings ((**a**): with 5% CeO_2_; (**b**): with 8% CeO_2_).

The SEM surface morphology and elemental distribution of the as-sprayed AT3 + 1%CeO_2_, AT3 + 5%CeO_2_, AT3 + 8%CeO_2_ coatings are shown in Figure 1. Element distribution mappings reveal that Al interdigitated with Ti, and the coating generated some crevices at the junction of two elements in the coating, which is one of the sources of pores or crevices in the as-sprayed coating; the cross-sectional photo in Figure 2 shows it more clearly.

During the coating spraying, the addition of CeO_2_ to the powder sufficiently aggravated the melting process of the powder, deposited at the pores or crevices of the coating, and formed a dense coating microstructure [21,22].

The melting point of rare-earth oxides is lower than that of A1_2_O_3_ and TiO_2_ [23]. Molten CeO_2_ particles initially precipitate and collide with molten A1_2_O_3_/TiO_2_ particles to form ceramic coatings during cooling and nucleation [24]. As a result, CeO_2_ may be employed as the non-spontaneous nucleation core of ceramic coatings, increasing nucleation rates and refining coating grains. Simultaneously, the chemical activity of the Ce element is quite high, which can affect the nucleation process of the coated grains, play a role in grain purification, and preferentially precipitate at the grain boundary. Therefore, the elemental mapping revealed a higher concentration of Ce at the interfaces, and the pores or crevices of the coating were filled.

In comparison to the distribution of Ce in the coatings with different CeO_2_ concentrations, when 1% CeO_2_ was added to the coatings, the distribution of Ce was significantly diffused. The Ce was more concentrated on the surface pores or crevices of the coating and the interface where the Al-Ti components interacted when the CeO_2_ content reached 5%. When the concentration is increased to 8%, Ce became enriched in the gaps and coexisted more with Ti, as shown in Figure 2.

### 3.2. XRD Patterns

**Figure 3:**
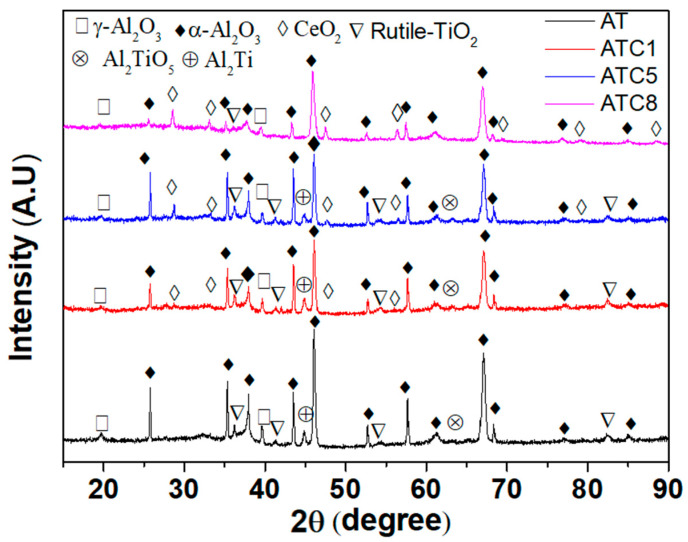
XRD patterns and phase analysis of the pure AT3 and doped with 1%,5%,8% CeO_2_ coatings.

Figure 3 shows XRD patterns of the pure AT3 and doped with 1%, 5%, and 8% CeO_2_ coatings produced by plasma spraying. The spectra reveal that rutile-TiO_2_ and α-Al_2_O_3_ made up the majority of the AT3 coating, with tiny amounts of γ-Al_2_O_3_, Al_2_Ti, and Al_2_TiO_5_. When 1% CeO_2_ was added to the AT3 coating, the diffraction peak of CeO_2_ appeared in the spectra, which presented main diffraction peaks for CeO_2_ at 28.55° and 47.485°. Moreover, the diffraction peak of Al_2_TiO_5_ decreased, and the intensity of the diffraction peak for Al_2_TiO_5_ became less intense.

The intensity of CeO_2_′s diffraction peak considerably increased with the addition of CeO_2_ to the coating. When the addition of CeO_2_ increased to 8%, the diffraction peak of rutile-TiO_2_ in the pattern disappeared, and the primary components were α-Al_2_O_3_ and CeO_2_. Meanwhile, the diffraction peak intensity of α-Al_2_O_3_ was the greatest among the four coatings.

The addition of rare-earth oxides CeO_2_ reduced the content of γ-Al_2_O_3_ in the coating, but excessive additions occupied the “position” of TiO_2_, and prevented the formation of the AlTi phase, such as Al_2_Ti and Al_2_TiO_5_, which affected the performance of the coating.

### 3.3. XPS

**Figure 4:**
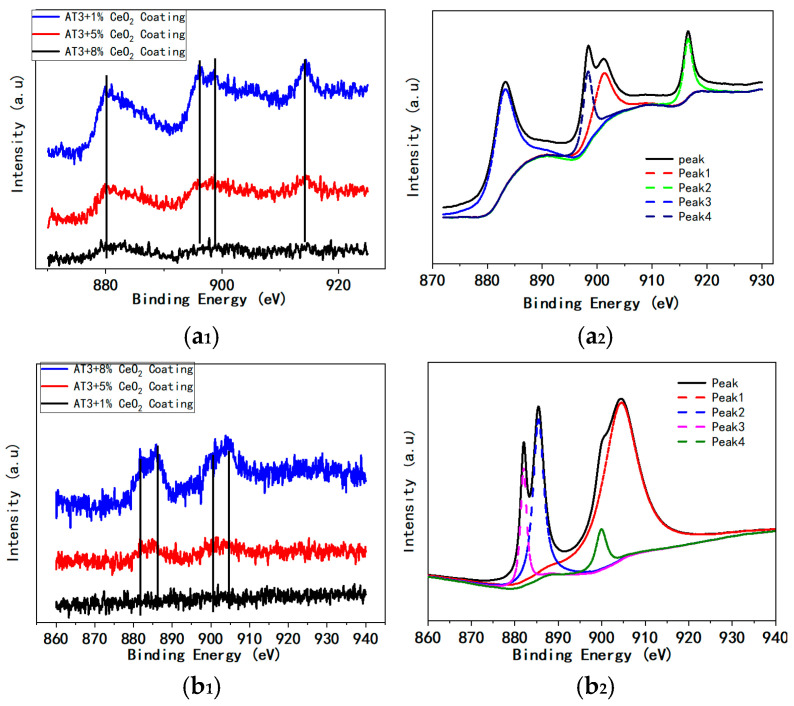
Three-dimensional XPS spectra of Ce and peak deconvolution in the coatings. a: as-sprayed coating; (**a_1_**): XPS patterns of the pure AT3 and doped with 1%, 5%, and 8% CeO_2_ coating; and (**a_2_**): Fitting result of Ce 3d pattern of AT3 + 8% CeO_2_ coating. b: The coating after 36 h immersion in 5% NaCl; (**b_1_**): XPS patterns of the pure AT3 and doped with 1%, 5%, and 8% CeO_2_ coating; and (**b_2_**): Fitting result of Ce 3d pattern of AT3 + 8% CeO_2_ coating.

Figure 4a shows the 3d XPS spectra of Ce in the as-sprayed coatings of AT3 + 1% CeO_2_, AT3 + 5% CeO_2_, and AT3 + 8% CeO_2_, where in Figure 4(a_1_) depicts the Ce3d spectra of the coating with various Ce additions, and Table 3 shows the intensity and deconvoluted peaks. There are two stable oxidation states of Ce (Ⅳ) and Ce (Ⅲ), and the electronic structure of Ce is 4f15d16s2. Ce (IV) and Ce (III) can be reversibly transformed by redox reaction with the amount of oxygen in the external environment, and the missing oxygen occurs in the form of an oxygen vacancy defect [25,26].

In Figure 4(a_1_), the spectra of Ce3d show four characteristic peaks, which are present at 883 eV, 898 eV, 901 eV, and 916 eV. Peak 916.3 eV is due to Ce (IV). The position of the characteristic peak Ce in the 1% CeO_2_ coating is clearly shifted, which should be related to Ce distribution and content. The intensity of each Ce peak increases clearly as the Ce concentration increases.

The fitting results of 3d XPS spectra of Ce in the AT3 + 8% CeO_2_ coating are shown in Figure 4(a_2_). The peaks can be fitted using four peaks, which correspond to Ce 3d3/2 and 3d5/2. There is no typical Ce (III) peak in the spectrum, indicating that Ce exists stably in the coating as Ce (IV) during thermal spraying, which is consistent with the XRD results.

Figure 4b shows the 3d XPS spectra of Ce in the coatings of AT3 + 1% CeO_2_, AT3 + 5% CeO_2_, and AT3 + 8% CeO_2_ after immersion for 36 h. Figure 4(b_1_) depicts the Ce3d spectra of the coating with various Ce additions after immersion for 36 h, and the results of the intensity and matching distinctive peaks are shown in Table 3.

In Figure 4(b_1_), the spectra of Ce3d for the coatings show four characteristic peaks, which are present at 882 eV, 885 eV, 899 eV, and 904 eV. By comparing the results of the as-sprayed coating, the position of the characteristic peaks for Ce3d changes; the characteristic Ce(IV) peak at 916 eV disappears, and a Ce(III) peak emerges at 885 eV.

The fitting results of 3d XPS spectra of Ce in the AT3 + 8% CeO_2_ coating after immersion for 36 h are shown in Figure 4(b_2_). There was a typical Ce (III) peak at 885 eV in the spectrum, indicating that a certain amount of Ce (IV) was converted to Ce (III) during long-term immersion. This indicates that during the long-term immersion, the rare-earth element Ce in the coating reacts with the corrosive medium in the solution, having a major impact on the coating’s corrosion resistance.

### 3.4. Open Circuit Potential Curve of Long-Term Immersion

**Figure 5:**
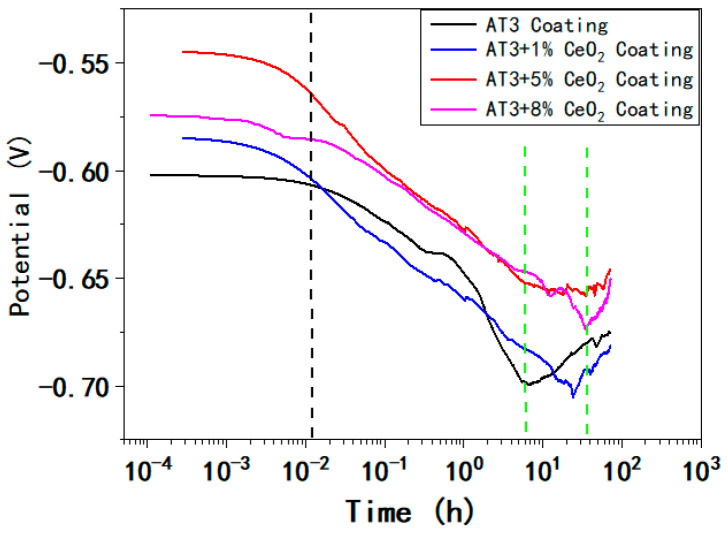
Open circuit potential (Eocp) vs. time curve of AT3, AT3 + 1%CeO_2_, AT3 + 5%CeO_2_, and AT3 + 8%CeO_2_ coatings immersed in 5% NaCl solution for 72 h (optimum data from 5 repeated experiments).

The open circuit potential (Eocp) vs. time curve of AT3, AT3 + 1% CeO_2_, AT3 + 5% CeO_2_ and AT3 + 8% CeO_2_ coatings immersed in 5% NaCl solution for 72 h is shown in Figure 5 As can be seen, the Eocp of AT3 + 5% CeO_2_ coating is always higher than that of the other coatings, while the Eocp of AT3 coating is the lowest during the initial phase.

The corrosion process of all the coatings immersed in 5% NaCl solution can be divided roughly into three phases. The initial phase is in the range of 0–50 s, when the potential shows a relatively gentle downward trend; the intermediate phase is from 50 s to 7–9 h, and the Eocp rapidly decreases; and in the last phase, the Eocp begins to rise.

The duration of the intermediate phase differs between coatings. After about 7 h of immersion, the potential of the AT3 coating dropped sharply to about −0.699 V. The potential began to move forward gradually with time, and there was a clear inflection point in the overall curve. The inflection point of the Eocp vs. time curve for the coating doped with CeO_2_ was then obviously extended. At around 23.8 h, the obvious inflection point of the Eocp vs. time curve for AT3 + 1%CeO_2_ coating appeared. At this point, the lowest value of Eocp was −0.7052 V, and the open circuit potential (OCP) gradually began to rise. At around 33 h, the Eocp curve of the AT3 + 5%CeO_2_ coating exhibited a distinct inflection point, and the OCP showed a relatively gentle transition.

At this point, the potential fluctuates between −0.657 V and −0.659 V before it began to rise at around 50 h. At around 33 h, the obvious inflection point of the Eocp vs. time curve for AT3 + 8%CeO_2_ coating appeared. The lowest value of Eocp was −0.7028 V, and the OCP gradually began to rise.

The three phases of the Eocp vs. time curve are associated with long-term immersion processes. At the beginning of the immersion process, the coating surface was infiltrated and wet by solution. The intermediate phase was the process where corrosive ions penetrated and corroded the coating; the Eocp value decreased with time, and the Eocp vs. time curve demonstrated a linear relationship. Because the surface of AT3 coating had more pores, the solution penetrated and corroded the surface of AT3 coating more easily. After doping with CeO_2_, the coating’s compactness increased, while its porosity decreased. The time required for the solution to penetrate and corrode the coating was relatively prolonged, and the Eocp fluctuated slightly. With the extension of immersion time in the latter phase, the OCP began to rise significantly. It suggests that corrosive ions have gotten through the coating and corroded the metal substrate.

### 3.5. Potentiodynamic Polarization

**Figure 6:**
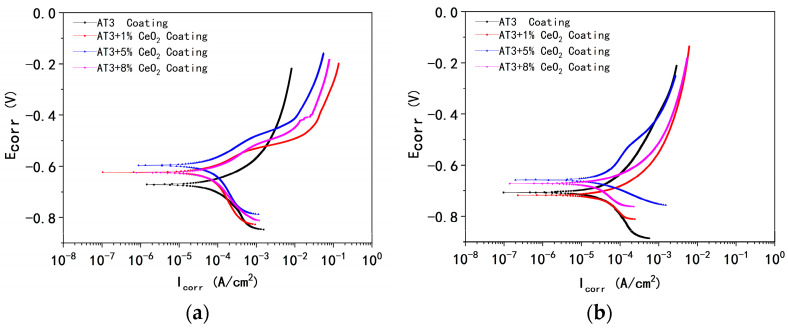
Potentiodynamic curve of AT3, AT3 + 1%CeO_2_, AT3 + 5%CeO_2_, and AT3 + 8%CeO_2_ coatings immersed in 5% NaCl solution ((**a**): immersed for 0.5 h; (**b**): immersed for 36 h) (optimum data from 5 repeated experiments).

**Figure 7:**
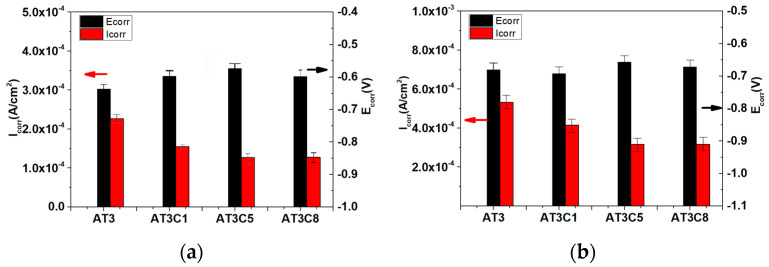
Corrosion potential and Tafel fitting results (mean ± SD, n = 5) for AT3, AT3 + 1%CeO_2_, AT3 + 5%CeO_2_ and AT3 + 8%CeO_2_ coatings immersed in 5% NaCl solution ((**a**): immersed for 0.5 h; (**b**): immersed for 36 h) (optimum data from 5 repeated experiments).

Figure 6 shows the potentiodynamic curve of AT3, AT3 + 1%CeO_2_, AT3 + 5%CeO_2_, and AT3 + 8%CeO_2_ coatings immersed in 5% NaCl solution for 0.5 h and 36 h. Figure 7 shows the corrosion potential and Tafel fitting results.

The results of potentiodynamic polarization measurement show that the corrosion potential of AT3 was the highest corrosion potential at the beginning of ± the experiment, and the corrosion rate of AT3 was the largest among the other CeO_2_-doped coatings. The corrosion current density of the coating after doping with 1% CeO_2_ was 1.55 ± 0.19 × 10^−4^ A/cm^2^, which is lower than that of the coating without CeO_2_. The corrosion current also decreased as the amount of CeO_2_ was increased. However, when doped with 8% CeO_2_, the corrosion rate was similar to that of the coating doped with 5% CeO_2_.

The results of the potentiodynamic polarization measurement changed after being immersed for about 36 h. At this time, the corrosion potential of the AT3 coating was lower than that of the AT3 + 1%CeO_2_ coating, but its corrosion potential density was slightly higher than that of the AT3 + 1% CeO_2_ one. The corrosion potential of the coating with 5% CeO_2_ was lower than that of other coatings, which is consistent with the open circuit potential measurement results. The corrosion current density of 5% CeO_2_ coating was slightly lower than that of 8% CeO_2_ coating, but they were very close.

### 3.6. EIS

**Figure 8:**
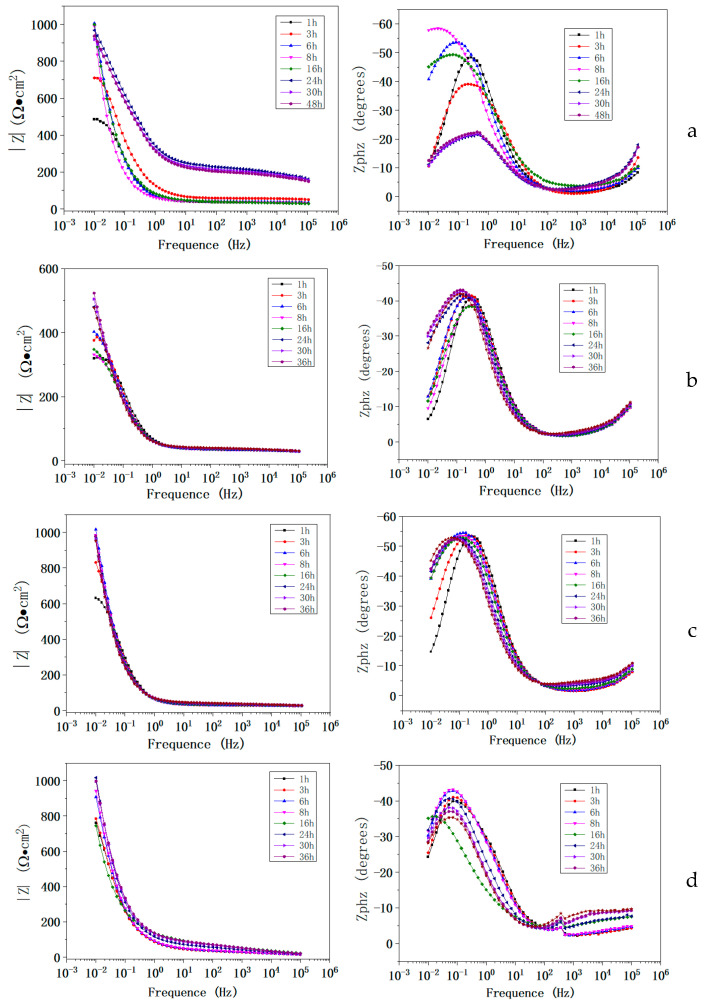
EIS curve of AT3, AT3 + 1%CeO_2_, AT3 + 5%CeO_2_, and AT3 + 8%CeO_2_ coatings immersed in 5% NaCl solution ((**a**): AT3 coating; (**b**): AT3 + 1%CeO_2_ coating; (**c**): AT3 + 5%CeO_2_ coating; (**d**): AT3 + 8%CeO_2_ coating) (optimum data from 5 repeated experiments).

The coatings with and without CeO_2_ were immersed in 5% NaCl solution, and the electrochemical impedance spectrum was measured at various times. Figure 8 shows the frequency–modulus and frequency–phase angle curves.

**Figure 9:**
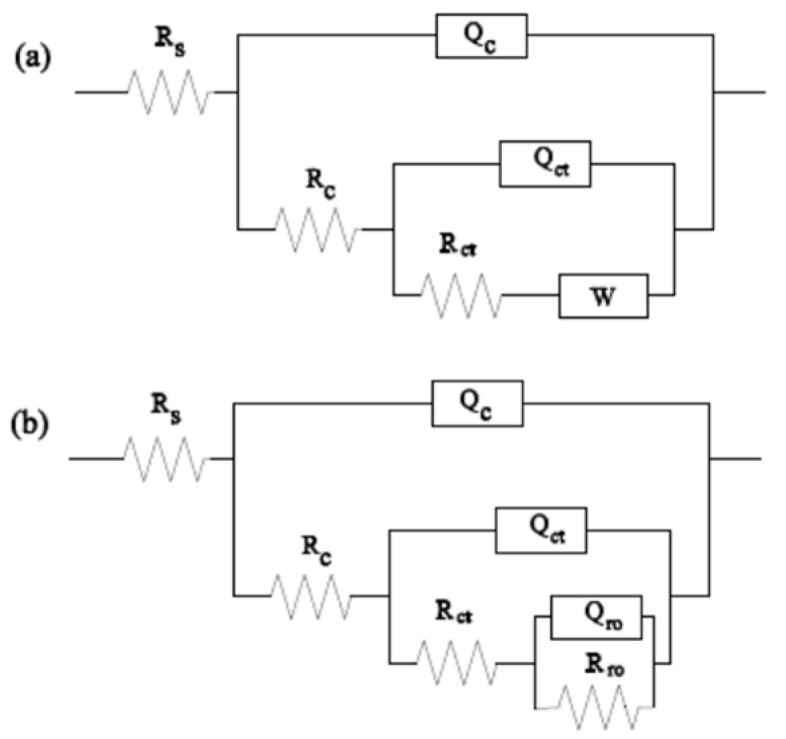
Equivalent electrical circuit for EIS result ((**a**): AT3 coating; (**b**): AT3 + 1%CeO_2_ coating, AT3 + 5%CeO_2_ coating, and AT3 + 8%CeO_2_ coating).

The equivalent electrical circuit shown in Figure 9 was used to fit the EIS results, in which the frequency–phase angle of AT3 coating was obviously different from that of other coatings. Therefore, the EIS result of the AT3 coating was fitted with (a), and the other coatings were fitted with (b) in Figure 8.

The electrolyte resistance (Rs) is connected in series with the coating unit system in Figure 9. Q_C_ is the phase angle element of the capacitor; Q_ct_ is the coating’s coated double layer element; and Q_ro_ is the phase angle element formed by doping with rare-earth elements CeO_2_. All of them reflect the inhomogeneity of the coating-substrate interface and are related to the electric double layer between the coating and the substrate [27,28].

R_c_ is the coating resistance, which is proportional to the coating thickness; R_ct_ is the coating diffusion resistance; and R_ro_ is the rust resistance caused by the addition of rare-earth elements. Z_w_ is the diffusion impedance; the angular frequency *ω* affects Z_w_’s value, which is related to the coating performance.

Z_w_ = (*jω*) ^−0.5^ Y_w_^−1^ (1)

where Y_w_ is a constant and j is an imaginary unit. The higher the Y_w_ value, the smaller the diffusion resistance of the medium. Diffusion resistance R_ct_, phase angle element of the coating caused by the addition of the rare-earth element, and Q_ct_ connected in series to reflect the coating characteristics when corrosive ions pass through the pores as a result of the adsorption of the intermediate products in the coating.

**Figure 10:**
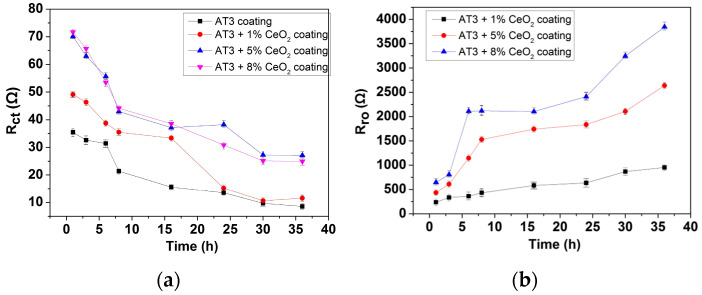
Main parameters (Rct (**a**) and Rpro (**b**)) of fitted electrochemical parameters (error bars represent standard deviation from quintuplicate tests; optimum data from 5 repeated experiments).

The fitting results have a mean square error of x^2^ < 10^−3^, and a relative error of less than 10% for each parameter. Figure 10 shows the main parameters of the fitted electrochemical parameters.

Figure 10a shows the curve of R_ct_ of four different coatings with immersion time. R_ct_ denotes the coating diffusion resistance in the equivalent electrical circuit, and the corrosion rate is generally inverse. According to the fitting results, the R_ct_ of AT3 coating was relatively higher than that of the others. The diffusion resistance of AT3-CeO_2_ coating significantly reduced with CeO_2_ doping, which is related to the reduction in coating porosity and defects and contributed to the current flow during the corrosion process [29].

R_ct_ of the coatings doped with 5% and 8% CeO_2_ was very close at the start of the experiment, matching the results of polarization curves. R_ct_ gradually decreased as immersion time increased, indicating that the corrosive medium began to penetrate the pores of the coating, reducing corrosion resistance and gradually increasing the corrosion rate. When immersion time reached 30 h, R_ct_ did not decrease, and the corrosion rate reached a steady state.

Rare-earth elements added to the coating have a certain chemical activity. With the action of corrosive medium, the resistance of rare-earth element R_ro_ became more apparent with the addition of corrosive medium, which contributed to the adsorption of corrosive medium and affected the corrosion behavior. The R_ro_ of coatings with 8% Ce was significantly higher than that of the other two coatings, indicating that the addition of excessive Ce increased the coating activity and adsorbed more chloride ions, but easily increased the corrosion rate of the coating in solution.

### 3.7. Discussion

According to the above experimental results, with the addition of CeO_2_ during the spraying, the coating has a significant effect on reducing the porosity and changing the phase structure of the coating. The element distribution mapping reveals that CeO_2_ is distributed in the coating’s flaws, cracks, and layer edges, filling the gaps created during spraying. All of these have improved the corrosion resistance of the coating, so as to delay the penetration of corrosive media, rather than providing intrinsic electrochemical protection.

During the long-term immersion of the coating, the reaction equation for the corrosion of AT3 coating under the standard concentration of NaCl is as follows:

(1) Hydrolysis reaction of Al_2_O_3_:

Al_2_O_3_ + 3H_2_O → 2Al^3+^ + 6OH^−^  ΔG_1_ = +397.8 kJ/mol (2)

(2) Coordination of Al^3+^ with Cl^−^

Al^3+^ + 4Cl^−^ → AlCl_4_^−^  ΔG_2_ = +322.5 kJ/mol (3)

The calculated ΔG_1+2_ = 719.3 kJ/mol > 0 shows that the AT3 coating has good corrosion resistance in the NaCl solution, and the potential–pH diagram also shows that Al_2_O_3_ is stable (passivation range) between pH 4–9, while in the more acidic or alkaline region, aluminum is dissolved in ionic form.

In the neutral NaCl solution, the concentration of chloride ions in the solution is 0.85 M. If the concentration of Al^3+^ is very low (such as 1 × 10^−6^ m), reaction 1 and reaction 2 need to be corrected as follows:

Δ*G*_2_ = Δ*G*_2_ + *R T* ln*Q*, *Q* = [Al^3+^] [Cl^−^]^4^/[AlCl_4_^−^] (4)

After correction, the still Δ*G*_2_ = 89.9 kJ/mol > 0. However, under non-ideal conditions (e.g., localized [Cl^−^] > 1 M, pH < 4 in defects of the coating, such as pores or crevices), the corrected Δ*G*_2_ (Equation (4)) approaches +89.9 kJ/mol, still positive but less prohibitive.

TiO_2_ in the coating reacts as follows in neutral NaCl solution:

The chemical properties of TiO_2_ are stable in neutral solution, and weak hydrolysis may occur.

TiO_2_ + 2H_2_O → Ti(OH)_4_  G = +63.5 kJ/mol (5)

The reaction is not spontaneous, and TiO_2_ is stable in neutral solution and does not participate in a significant corrosion reaction.

However, when there are pores or defects in the coating, Cl^−^ intrusion occurs locally, and the metal ions enriched in the pores are hydrolyzed. The pH may be reduced due to hydrolysis reaction (such as Al^3+^ hydrolysis to produce h^+^), leading to acidification (local pH reaches 2–3), promoting the dissolution of alumina and further corrosion of metal. In an environment with high chloride ion concentration, local corrosion is more likely to occur. Further calculation shows that when the pH of the local zone of the coating reaches 2.5 and the chloride ion concentration is 3M, Δ*G*_2_ (Equation (4)) approaches −28.3 kJ/mol.

When CeO_2_ exists in the pores or crevices of the coating, the long-term immersion process of CeO_2_ in the coating has an impact on the corrosion process.

Combined with the Ce-H_2_O phase diagram in the figure, the reaction of CeO_2_ in neutral NaCl solution is as follows:

(1) Hydrolysis of cerium oxide, the reaction is a dynamic equilibrium process, and the solubility of CeO_2_ is very low (KSP ≈ 10^−28^), but the trace dissolved Ce^4+^ may participate in the subsequent reaction.

CeO_2_ + 2H_2_O ⇌ Ce^4+^ + 4OH^−^ (6)

(2) Conversion of tetravalent Ce^4+^ to trivalent Ce^3+^

Ce^4+^ + *e*^−^ → Ce^3+^ (E° ≈ 1.28 V vs. SHE)  ΔG_1_ = −174 KJ (7)

(3) Hydrolysis of Ce^4+^

Ce^3+^ + 3OH^−^ → Ce(OH)_3_↓ (pH > 6)   ΔG_2_ = −285 kJ/mol (8)

(4) Hydrolysis of Ce^4+^

Ce^4+^ + 4OH^−^ → Ce(OH)_4_↓ or CeO_2_ nH_2_O  ΔG_3_ = −320 kJ/mol (9)

A compact Ce (OH)_3_/Ce (OH)_4_ film was formed to cover the metal surface. The micropores of the coating were blocked, and the penetration rate of Cl^−^ was reduced.

So, we assume that CeO_2_ may prefer to remain solid and release a small amount of Ce^3+^or Ce^4+^, while Ce^3+^or Ce^4+^ will hydrolyze to form Ce(OH)_3_ or Ce(OH)_4_ precipitation at neutral pH solution, which may serve as a protective layer. As shown in the XPS results, Ce (Ⅳ) and Ce (Ⅲ) were formed during the corrosion process of the coating. The change in Ce valence state may be accompanied by a local volume effect, further sealing the pores and indirectly strengthening the barrier function.

The local acidification in the pores of the coating results in high hydrogen ion concentration and low pH value, which also promotes the conversion of Ce. In particular, with the strong permeability of chloride ions, the conversion process of local Ce ions in the pores will be accelerated, resulting in the generation of more Ce^3+^.

Therefore, CeO_2_ in the coating can significantly improve the local corrosion caused by coating defects during long-term immersion. However, the porosity in the coating is limited. Once the amount of CeO_2_ reaches the threshold, more CeO_2_ tends to aggregate in the coating, forming Ce-rich regions.

CeO_2_ + 4Cl^−^ + 2H_2_O → CeCl_4_ + 4OH^−^  ΔG = −403.8 kJ/mol (10)

CeO_2_ + 3Cl^−^ + 2H_2_O → CeCl_3_ + 4OH^−^  ΔG = −604.6 kJ/mol (11)

On the other hand, the CeO_2_ in the coating also reacts with higher concentrations of Cl^−^ in the solution during long-term immersion, as follows:

The CeO_2_ in the coating spontaneously reacts with chloride ions in the solution, becoming the preferred area for corrosion; this is similar to the EIS measurement results. As corrosion occurs in these areas, local pH surpasses the stability range (passivation range) of Al_2_O_3_ coating, which leads to the coating corrosion. Notably, when the CeO_2_ content in the coating is high, localized enrichment of cerium oxide occurs. Specifically, an optimal filling effect on coating gaps and defects is achieved with a 5% addition of CeO_2_. When it exceeds 5%, the enriched CeO_2_ is more likely to react with chloride ions. When calculating the ΔG for coatings with 5% and 8% CeO_2_, 5% CeO_2_:ΔG5%CeO_2_ ≈ −180 kJ/mol; 8% CeO_2_: ΔG8%CeO_2_ ≈ −220 kJ/mol. It was observed that the ΔG was lower at 8%, indicating a higher propensity for corrosion reactions to occur.

As can be seen from Figure 10a, which is the figure of the evolution of charge transfer resistance (Rct) for coatings with different CeO_2_ content, the coating doped with CeO_2_ (5%) shows high initial Rct (about 71 kΩ·cm^2^) and slower degradation during immersion, and with excessive doped CeO_2_ (8%), rapid Rct decline emerges. The Rct trends were directly governed by CeO_2_-induced microstructural and electrochemical changes. The excessive CeO_2_ (8%) at the coating defects induced the spontaneous Ce-Cl reactions under defect conditions (ΔG8% CeO_2_ ≈ −220 kJ/mol). These reactions generated highly soluble CeCl_3_/CeCl_4_, establishing ion-conduction pathways that reduced Rct. Figure 10b further demonstrates that increasing CeO_2_ content correlated with a significant rise in electrochemically active sites across the coating. The XPS analysis further confirmed that excessive cerium oxide promoted significant Ce^3+^ formation during long-term immersion, demonstrating redox-driven corrosion acceleration.

**Figure 11:**
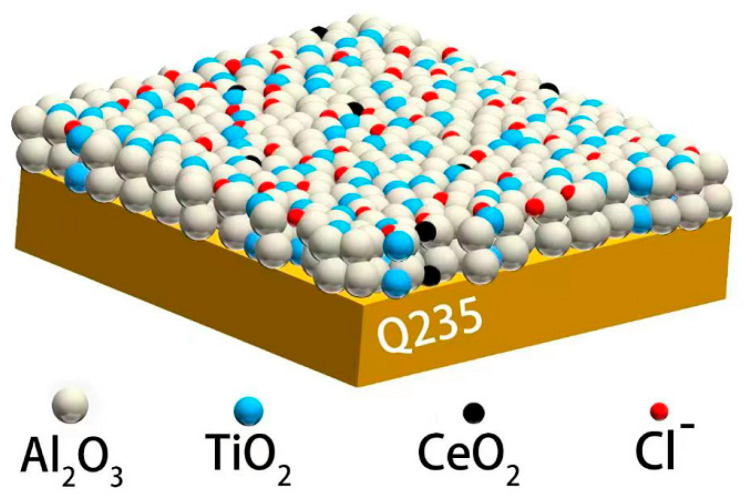
Diagram of corrosion process of coating in 5% NaCl solution.

As shown in Figure 11, the addition of CeO_2_ to the coating can fill the defect areas of the coating, physically hindering the infiltration of corrosive ions. Thus, we hypothesize that the Ce element has high chemical activity and is prone to react preferentially with corrosive ions, which is more evident during long-term immersion. Excessive additions of CeO_2_ in the coating will adsorb corrosive ions and react to produce Ce^3+^ in the solution. It was widely assumed that the stability of Ce^4+^ is greater than that of Ce^3+^ [30]. The Ce_2_O_3_ generated during the long-term corrosion process boosts the activity of the coating and increases corrosive ion adsorption. As a result, high Ce has no discernible effects on corrosion, but the opposite effects. Scalability remains constrained by powder agglomeration during mixing. Achieving <5% agglomeration (Section 2) requires stringent process control, increasing production costs by ~30%.

Limitations of this study include the lack of surface hydrophobicity data (contact angle) and FTIR analysis, which could further elucidate the wetting behavior and chemical bonding changes during immersion. Future work will incorporate these characterizations to provide a more comprehensive understanding.

## 4. Conclusions

The long-term immersion corrosion behavior of Al_2_O_3_-TiO_2_ coating doped with 1%, 5%, 8% CeO_2_, and without CeO_2_, prepared by plasma spraying, was investigated in 5% NaCl solution. XPS, SEM, and electrochemical methods were used to study the impact of different CeO_2_ on the corrosion process. The results were as follows:

1. The corrosion resistance of CeO_2_-doped coatings was significantly superior to the undoped coating during long-term immersion, primarily due to reduced porosity. CeO_2_ addition also decreased the γ-Al_2_O_3_ content. However, at 8% CeO_2_, excessive oxide occupied interstitial spaces, displacing TiO_2_ and inhibiting the formation of beneficial AlTi phases (Al_2_Ti, Al_2_TiO_5_), degrading coating performance.

2. The coating doped with 5% CeO_2_ demonstrated the optimal corrosion resistance among all samples, indicating that this concentration provides effective pore filling.

3. The XPS analysis confirmed the conversion of Ce(IV) to Ce(III) during long-term immersion, highlighting the high chemical activity of Ce within the coating. This activity, coupled with thermodynamic calculations, revealed that higher CeO_2_ content enhances corrosive ion adsorption. Consequently, rare-earth oxide doping levels exceeding 5% actively participate in the corrosion process rather than acting solely as passive inhibitors. And the cost of raw materials increased by 30%. Large-scale production also needs to control the powder agglomeration rate <5%.

4. Limitations of the research include (i) due to a lack of surface hydrophobicity data (contact angle) and surface chemistry, the possible mechanisms are still hypothesized and inferred, (ii) cost increase (~30%) from rare-earth doping, and (iii) agglomeration control requirements for industrial scale-up.

## Figures and Tables

**Table 1:** Proportion of powder composition for plasma spraying (mass fraction,%).

Specimens	AT3/wt%	CeO_2_/wt%(Add as Powder)	Ce/wt%
1	100	0	0
2	99	1	0.81
3	95	5	3.87
4	92	8	6.03

**Table 2:** Plasma spray process parameters.

Voltage/V	Electric Current/A	Spray Distance/mm	Ar Flow Rate/(L·min^−1^)	H_2_ Flow Rate/(L·min^−1^)	Powder Feeding/(g·min^−1^)
27	580	100	45	12	30

**Table 3:** XPS spectra fitting results for Ce 3d of AT3 + CeO_2_ coatings before and after immersion.

	As Prepare					After Immersion				
	Peak	Binding Energy (eV)	Intensity (a.u)	FWHM (eV)	Valence State	Peak	Binding Energy (eV)	Intensity (a.u)	FWHM (eV)	Valence State
AT3 + CeO_2_ 1% Coating	1	881.92	7109.97	3.75	Ce^4+^	1	881.71	389.85	0.89	Ce^4+^
	2	899.74	3437.79	4.07	Ce^4+^	2	885.46	817.29	2.83	Ce^3+^
	3	904.14	2461.44	3.95	Ce^4+^	3	904.01	84.32	1	Ce^4+^
	4	916.65	2196.46	3.2	Ce^4+^	-	-	-	-	-
AT3 + CeO_2_ 5% Coating	1	883.01	29,485.89	4.45	Ce^4+^	1	881.71	3731.99	3.71	Ce^4+^
	2	898.26	9825.02	2	Ce^4+^	2	885.23	5326.55	4.43	Ce^3+^
	3	901.05	14,137.62	4.06	Ce^4+^	3	900.07	4005.15	4.86	Ce^4+^
	4	916.49	7491.32	1.88	Ce^4+^	4	904.13	4776.94	6.29	Ce^4+^/Ce^3+^
AT3 + CeO_2_ 8% Coating	1	883.03	29,914.54	4.52	Ce^4+^	1	882.02	4514.66	1.69	Ce^4+^
	2	898.28	10,104.11	2.03	Ce^4+^	2	885.44	10,634.82	2.92	Ce^3+^
	3	901.08	13,584.21	3.95	Ce^4+^	3	899.88	2177.49	2.99	Ce^4+^
	4	916.51	7339.72	1.85	Ce^4+^	4	904.34	33,682.54	9.41	Ce^4+^/Ce^3+^

## Data Availability

The original contributions presented in this study are included in the article. Further inquiries can be directed to the corresponding author.
